# Periodontal Health and Systemic Conditions

**DOI:** 10.3390/dj8040130

**Published:** 2020-11-19

**Authors:** Glendale Lim, Upasna Janu, Lan-Lin Chiou, Kaveri Kranti Gandhi, Leena Palomo, Vanchit John

**Affiliations:** 1 Department of Periodontology, Indiana University School of Dentistry, Indianapolis, IN 46202, USA; glenlim@iu.edu (G.L.); ujanu@iu.edu (U.J.); lchiou@iu.edu (L.-L.C.); kkgandhi@iu.edu (K.K.G.); 2 Department of Periodontology, Case Western University, School of Dentistry, Indianapolis, IN 46202, USA; leena.palomo@case.edu

**Keywords:** periodontitis, systemic diseases, general health

## Abstract

According to the new classification proposed by the recent 2017 World Workshop on Periodontal and Peri-implant Diseases and Conditions, periodontitis, necrotizing periodontal diseases, periodontitis as a manifestation of systemic diseases, and systemic diseases or conditions affecting the periodontal supporting tissues, are considered as separate entities. Scientific evidence has demonstrated that periodontal diseases are not just simple bacterial infections but rather complex diseases of multifactorial complexity that interplay with the subgingival microbes, the host immune, and inflammatory responses. Despite dental plaque biofilm being considered the primary risk factor for periodontitis in the vast majority of patients that dentists encounter on a daily basis, there are other factors that can also contribute and/or accelerate pathologic progressive attachment loss. In this article, the authors aim to briefly review and discuss the present evidence regarding the association between periodontal diseases and systemic diseases and conditions.

## 1. Overview—Periodontal Disease Etiology and Pathogenesis

The interrelationship between diseases in the mouth and body as a whole is not a new concept as it has been debated for over a hundred years. The theory of “focal infection” was introduced by William Hunter as a way to connect oral infections or sepsis with infections in other parts of the body [1]. This led to the removal of many “infected teeth” with the hope that this would also lead to the cure of distant disease conditions. It was only in the 1980s and 1990s that there was concerted effort at gathering scientific evidence on the topic, namely, establishing links between chronic periodontal disease and other systemic diseases [2,3].

Periodontitis is the outcome of progressive and persistent inflammation of the supporting tissues of the teeth leading to clinical attachment loss, alveolar bone loss, and periodontal pocket formation. As has been clearly documented over the years, periodontitis is a multifactorial disease involving a microbial challenge, a host immune–inflammatory response, other local factors, and genetic factors. Periodontitis is a major public health problem due to its high prevalence of 42% of the U.S. population over 30 years old, being more prevalent amongst the elderly, particular races, and smokers [4]. The annual total expenditure in the U.S. on periodontal and preventive procedures exceeds USD 14 billion, with the majority of the spending focused on preventive procedures [5].

Starting with the debate in the 19th century between non-specific and specific plaque theories to the later experimental disease and longitudinal models, bacterial plaque and its byproducts in a susceptible host have been recognized as the primary etiology factor. Specific cultivated subgingival anaerobic red-complex bacteria (*P. gingivalis*, *T. forsythia*, and *T. denticola*) were identified to have a strong virulent effect in the initiation and perpetuation of disease in the periodontium [6]. Additionally, *P. intermedia* and *F. nucleatum* were found to be associated with chronic form of periodontitis [7], while *A. actinomycetemcomitans* has been shown to be associated with the more aggressive form of periodontitis [8]. It is the microbiota of the dental plaque biofilm that drive the host inflammatory response, which ultimately leads to tissue destruction. The hyper-host immune response results in increased pro-inflammatory mediator production such as cytokines, eicosanoids, kinins, complement activation products, and matrix metalloproteinases [9]. In the susceptible host with compromised defense mechanisms, the uncontrolled bacterial challenge alters the host–microbial crosstalk and leads to the destruction of epithelial and connective tissue [10].

Current scientific evidence has demonstrated that periodontal diseases are not just simple bacterial infections but rather complex diseases of multifactorial complexity that interplays with the subgingival microbes, the host immune, and inflammatory responses [11]. Accordingly, it is clear that bacterial infections and their associated host immune responses are involved in the pathogenesis of periodontitis.

## 2. The Link between Periodontal Diseases and Systemic Conditions

Despite dental plaque biofilm being considered the primary risk factor for periodontitis in the vast majority of patients that dentists encounter on a daily basis, there are other factors that can also contribute and/or accelerate the pathologic progressive attachment loss. One should also recognize that the periodontium may be considered as a reservoir of bacteria, bacterial products, and inflammatory and immune mediators which can interact with other organ systems remote from the oral cavity. In the 2012 joint European Federation of Periodontology (EFP) and the American Academy of Periodontology (AAP) consensus report, it concluded that periodontitis may induce systemic inflammation via translocated circulating oral microbiota that impacts upon the development of atherothrombogenesis, thus suggesting the biological interaction and association between periodontal diseases and cardiovascular disease [12].

Mechanisms by which chronic periodontal disease may add to systemic disease may be considered as being via a direct and an indirect route. The direct route is via ulceration in the lining of periodontal pockets, which can become a passage for bacteria into the systemic circulation leading to bacteremia that allows periodontal disease bacteria to settle in distant organs aggravating existing disease conditions. [13]. The indirect route posits that chronic periodontal disease, being a significant source of inflammation, may play a role in other disease conditions in which inflammation is a major component. The role of the C-Reactive protein in this pathogenesis appears to be one that has strong evidence in its favor [14,15]. A recent study, which used ligature-induced periodontal disease in mouse models and human experimental gingivitis model, demonstrated that periodontal inflammation can result in increased polymorphonuclear neutrophils (PMNs) and potentially prime the systemic innate immune response [16]. This PMN-mediated hyperinflammatory innate immune response could be the plausible biologic crosslink between periodontal disease and the other chronic inflammatory diseases such as cardiovascular diseases, diabetes and arthritis.

The association between periodontal health and disease along with some commonly encountered systemic conditions and habits will be reviewed in this article.

### 2.1. Disease Modifiers of Periodontitis

Risk factors for periodontal disease such as diabetes mellitus and smoking have been identified and confirmed through longitudinal studies. These systemic, environmental, and psychological factors do not directly cause periodontitis, but rather, modify the host inflammatory response that is characterized by an altered vascular and cellular response which then predisposes or accelerates the destruction of the periodontal tissues [17]. There are no specific periodontal phenotypic features that are solely unique to diabetic patients suffering from periodontitis compared to that for patients suffering with periodontitis alone. However, extensive evidence shows that chronic uncontrolled hyperglycemia affects periodontitis progression [18,19,20]. Similarly, there are no unique periodontal phenotypic features in smokers; however, it is well established that smoking increases the risk of periodontitis by four times compare to non-smokers [21,22]. It can be concluded that diabetes and smoking modify the severity of periodontal disease progression, making periodontitis more aggressive and resistant to treatment [23].

#### 2.1.1. Smoking

Cigarette smoke is a complex mixture of chemicals, containing more than 7000 chemical compounds [24]. Tobacco smoking has been identified as a risk factor for periodontitis [25]. It has been reported that smokers are 2.6 to 6 times more likely to exhibit periodontal destruction than nonsmokers [26] and the effects of smoking on periodontal disease exhibit a dose–response relationship [27]. Smoking adversely affects the periodontal tissues and/or the progression of periodontal disease through a variety of mechanisms. Increasing periopathogenic bacteria (e.g., *P. gingivalis*, *T. forsythia*, *T. denticola* and *A. actinomycetemcomitans*) in subgingival plaque, abnormal neutrophil chemotaxis and phagocytosis, elevated pro-inflammatory cytokines (e.g., TNF-α) and proteolytic enzymes (e.g., MMP-8) and alteration in fibroblast functions have been demonstrated in cigarette smokers [27,28]. On the other hand, electronic cigarettes (e-cigs), producing aerosol by heating a nicotine-containing solution, have been commercialized since 2003 [29]. The prevalence of e-cig use in the United States was 3.2% in 2018 based on the National Health Interview Survey. While the prevalence among adults did not change significantly from 2014 to 2018, increasing prevalence among adolescents and young adults was reported [30]. Nevertheless, e-cigs cannot be viewed as a safe alternative to conventional cigarettes [31]. In vitro studies have shown that e-cig aerosols can have detrimental effects on gingival epithelial cells and periodontal fibroblasts [31]. Although a cross-sectional study did not find significantly worse periodontal conditions in e-cig users than in never smokers [32], a recent study revealed that e-cig users have a higher risk of being diagnosed with gum disease and periodontal bone loss compared to subjects who have never used electronic nicotine delivery systems [33].

#### 2.1.2. Obesity, Metabolic Syndromes, and Diabetes Mellitus

Obesity, featured by excessive body fat accumulation, is a chronic disease which has been reported to be associated with numerous diseases, such as hypertension, coronary heart disease, type 2 diabetes and cancer [34]. Additionally, obesity can have a negative effect on periodontal health [35]. The increasing production of proinflammatory cytokines in adipose tissue is considered to be responsible for the mechanisms underlying these associations [36]. On the other hand, metabolic syndrome is a clustering of metabolic conditions, correlating with increased risk for cardiovascular disease and type 2 diabetes. Criteria for defining the metabolic syndrome include central obesity, hypertriglyceridemia, low high-density lipoprotein (HDL) cholesterol, hypertension and elevated fasting plasma glucose. Individuals presenting with at least three of these components are diagnosed with metabolic syndrome [37]. The prevalence of the metabolic syndrome varies worldwide, ranging from less than 10% to more than 40% [38]. Based on cross-sectional analysis, it was revealed that patients with moderate or severe periodontitis are more likely to have metabolic syndrome compared to subjects with no or mild periodontitis [39]. A cohort study also demonstrated that long-term exposure of metabolic syndrome is related to deep probing depths and alveolar bone loss [40]. Oxidative stress might be a bidirectional link between periodontitis and metabolic syndrome, with reduced insulin sensitivity, decreased antioxidant capacity and increased oxidative damage involved in the interaction [41].

Diabetes mellitus is a group of metabolic diseases characterized by hyperglycemia resulting from defects in insulin secretion, insulin action, or both. The chronic hyperglycemia of diabetes can result in long-term multi-organ damage and failure, especially affecting the eyes, kidneys, nerves, heart, and blood vessels [42]. Diabetes mellitus is characterized by the body’s inability to properly control glucose levels in the blood. The two main forms of diabetes mellitus that have been shown to have an impact on periodontium are type 1 and type 2. In Type I Diabetes, the β cells of the pancreas produce little to no insulin. Since these patients are generally managed with supplemental insulin, Type 1 Diabetes can also be called Insulin-Dependent Diabetes Mellitus (IDDM). In contrast, Type 2 Diabetes is characterized by the decreased number or defectiveness of insulin receptors and can therefore also be known as Non-Insulin-Dependent Diabetes Mellitus (NIDDM).

**Figure 1:**
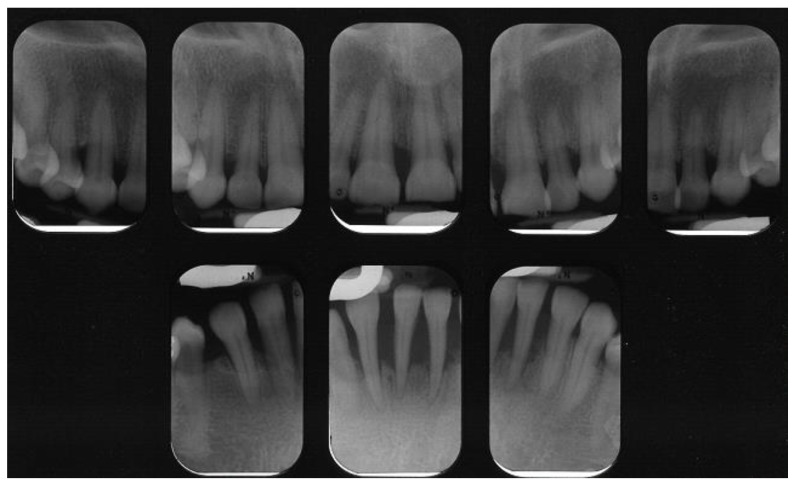
Peri-apical radiographs of patient with Type 2 Diabetes Mellitus. Severe loss of supporting bone is evident. Courtesy of Dr. Brittany Lane.

There is a strong evidence that diabetes is a risk factor for gingivitis and periodontitis, and the level of glycemic control appears to be an important determinant in this relationship [43]. Patients with elevated blood-glucose (≥120 mg/dL) had an odds ratio (OR) of 2.46 for having severe periodontal disease [44]. The poorly controlled diabetics demonstrated a higher amount of inflammation [45]; it is the only systemic condition with a positive association with attachment loss [46] (See Figure 1 for clinical case of a patient with Type 2 Diabetes Mellittus and severe periodontitis). The hyperglycemic environment leads to capillary basement membrane thickening, impaired oxygen diffusion and waste elimination. The hypofunction of neutrophils impairs the host defense mechanism and alters the overall immune function against infection [47]. Hyperglycemia changes fibroblast metabolism, inhibit osteoblastic cell proliferation and impaired osseous healing. The high blood glucose state also leads to production and accumulation of advanced glycation end products (AGEs). AGEs bind to monocytes and macrophages, causing them to release more pro-inflammatory cytokines such as IL-1β, TNF-α and PGE_2_, which lead to tissue destruction [48,49]. The systemic effects of diabetes contribute to periodontal disease through an increased inflammatory state, oxidative stress in the body, and decrease in the repair mechanisms (See Table 1 for summary of oral manifestations of diabetes mellitus; Table 2 for the EFP and AAP recommendation for managing diabetic patient). 

#### 2.1.3. Adverse Pregnancy Outcomes (APO)

The relationship between periodontal diseases and adverse pregnancy outcomes (APO) has been explored by researchers in the last two decades. Pregnant women with active periodontal diseases have been identified as being at risk for APO such as preterm birth (PTB), fetal growth restriction, low birthweight, pre-eclampsia and gestational diabetes [52]. Preterm low-birth weight (PLBW) cases were defined as a child born with a birth weight of less than 2500 g along with one or more of the following conditions: gestational age less than 37 weeks, preterm labor, or premature rupture of membranes. Epidemiological and intervention trial studies have suggested that mothers with active periodontal disease present with an independent risk factor for preterm low-birth weight. A case-control epidemiological study demonstrated the association through multivariate logistic regression models and showed that maternal periodontitis (defined as ≥60% of all sites with attachment loss of ≥3 mm) had an adjusted odds ratio of 7.9 for preterm low-birth weight babies [53]. This was the first study that demonstrated association between poor maternal periodontal condition and adverse pregnancy outcome. A 2007 meta-analysis study reported a likely association between PTB and/or PLBW (OR = 2.83, 95% CI: 1.95–4.10) [54].

Two plausible biological mechanisms, the direct and indirect pathways, have been proposed to explain the pathophysiological relationship between active maternal periodontal disease and APO [55]. The direct pathway describes the vertical transmission of periodontal pathogens (*P. gingivalis)* being disseminated through the umbilical cord and colonizing in the placenta which potentially contributes to placental dysfunction [56], whereas the indirect pathway describes the systemic circulation of the locally and systemically produced pro-inflammatory mediators leading to local inflammatory response at the feto-placental unit. As a sequela of periodontal disease, pro-inflammatory mediators such as interleukin-1 (IL-1, 6, 8), tumor necrosis factor (TNF-α) are produced. These mediators activate immune cascade responses to produce prostaglandin E2 (PGE2). As a result, the high level of PGE2 induce uterine contractions and increase the occurrence or APO [55].

### 2.2. Necrotizing Periodontal Diseases (NPDs)

**Figure 2:**
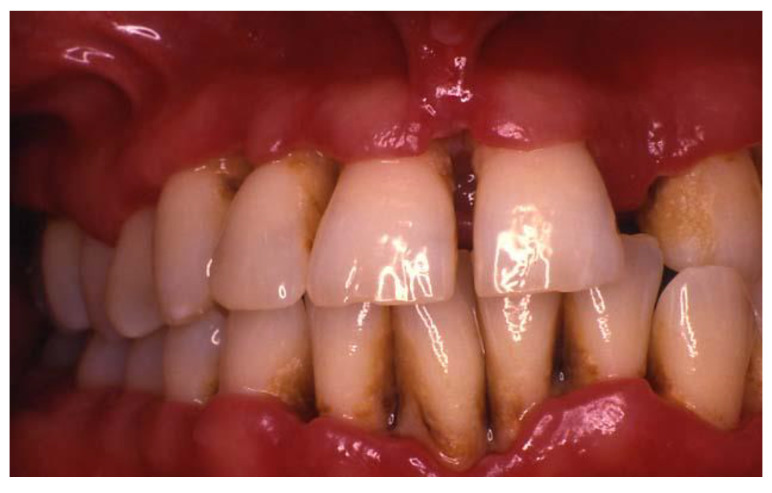
A 33-year-old patient presented with Generalized Necrotizing Periodontitis. Interdental papillary necrosis and ulceration of the periodontal pocket epithelium were evident. Courtesy of Dr. Vanchit John.

**Figure 3:**
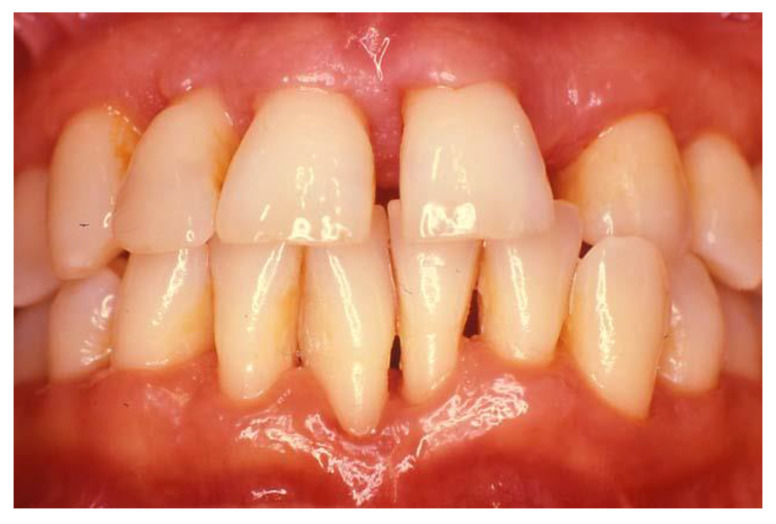
The patient with necrotizing periodontal disease (Figure 2) following 2 years of treatment following testing for HIV, initiating initial therapy followed by surgical treatment and correction of modifiable risk factors such as smoking. Courtesy of Dr. Vanchit John.

NPDs are infectious conditions that exist as a distinct diagnosis entity where bacteria and predisposing factors (such as malnutrition, psychological stress, tobacco and alcohol use) play a critical role in the disease pathogenesis. The typical NPD’s clinical presentation includes “punched-out” interdental papilla necrosis and ulcers, profuse gingival bleeding and acute inducible pain, pseudomembrane formation over the necrotic area, and halitosis [57,58] (See Figure 2 for clinical case presentation of a generalized necrotizing periodontitis case; Figure 3 for post-periodontal treatment of the same case). Extraoral findings and symptoms such as adenopathy and fever have also been reported in some cases [59]. In severe immunocompromised cases, especially in acquired immunodeficiency syndrome (AIDS) patients, sequestrum of interproximal bone may be present. The prevalence of NPDs in the general population ranges from 0.51 to 3.3% [58,60,61] and 0.3 to 11.1% in human immunodeficiency virus (HIV)/AIDS seropositive patients [62,63,64,65]. *Prevotella intermedia, Treponema, Selenomonas* and *Fusobacterium* species were considered as part of the pathogenic flora [66,67,68]. Similar to periodontitis, the etiology of NPDs is oral plaque; however, the compromised systemic condition of patients affected by NPDs allows for a sudden severe ulceration and necrosis of periodontal tissues that does not typically occur in the general healthy population [59].

### 2.3. Periodontitis as a Manifestation of Systemic Diseases

Rare genetic diseases (i.e., Papillon Lefevre Syndrome, Down syndrome, Leucocyte adhesion deficiency, etc.), metabolic and endocrine disorder (i.e., Hypophosphatasia, Glycogen storage disease), disease affecting connective tissues (i.e., Ehlers–Danlos syndrome, Systemic lupus erythematosus) can induce early attachment loss and tooth loss. For these conditions, described by Albandar et al., the main etiology of the attachment loss is the genetic disorder and not the classic multifactorial plaque-induced periodontitis [69]. Periodontal breakdown is primarily due to the systemic disease alone, and plaque control can only slow down its progression. For this type of periodontal disease, the attachment loss cannot be effectively treated unless the primary systemic condition that is causing periodontitis can be targeted. While there is weak evidence that plaque control may be beneficial in reducing the progressive attachment loss, genetic counseling and good plaque control are suggested to slow down the progression rate of periodontitis as manifestation of systemic diseases [69].

#### 2.3.1. Down Syndrome

Down syndrome (DS), also known as trisomy 21, is one of the most common congenital disorders. According to a recent report based on the data from the National Birth Defects Prevention Network, Down syndrome occurs in 1 in every 707 births in the United States [70]. Characteristics of patients with Down syndrome include mental deficiency and growth retardation; besides, high prevalence of periodontal disease and rapid progression of the disease have been reported [25,71]. The link between periodontal disease and Down syndrome can be attributed to various factors. While ineffective oral hygiene, presence of calculus and secondary local factors (e.g., malalignment of teeth) can play a role in the occurrence and progression of the disease, altered immune responses, such as impaired PMN leukocytes and monocytes chemotaxis, reduced capacity for PMN phagocytosis and defects in both T- and B-lymphocytes function, are considered as the main factors since the destruction of the periodontium cannot be solely explained by the bacterial plaque or other local factors [25,71].

#### 2.3.2. Human Immunodeficiency Virus (HIV)

The human immunodeficiency virus (HIV) is a retrovirus, which infects CD4^+^ T cells, bone marrow progenitor cells and developing thymocytes, thereby leading to immune dysfunction [72]. Acquired immunodeficiency syndrome (AIDS) is the last stage of HIV infection, diagnosed when CD4 cell count is less than 200 cells/mm^3^. HIV-associated periodontal lesions include linear gingival erythema, a distinct linear erythematous band limited to the free gingival margin [73] and necrotizing periodontal diseases (NPDs) [25]; in addition, periodontitis patients who are infected with HIV exhibit increased gingival recession and periodontal attachment loss [74]. Compromised immune system along with the change of microbial composition account for the disease process. In a clinical study, high levels of spirochetes, yeasts and herpes-like viruses were observed in HIV-positive patients with necrotizing periodontitis [75]. Opportunistic microorganisms, particularly candida species, might play a crucial role in the development of periodontal lesions observed in HIV-infected patients [74].

#### 2.3.3. Rheumatoid Arthritis (RA)

Rheumatoid arthritis and periodontitis share similar pathobiology. Rheumatoid arthritis is a chronic autoimmune destructive inflammatory disease affecting periarticular tissue and bone characterized by the accumulation and persistence of an inflammatory infiltrate in the synovial membrane which then can lead to synovitis and the destruction of the joint architecture [76]. Individuals suffering from advanced rheumatoid arthritis are more likely to experience moderate to severe periodontitis compared to non-rheumatoid arthritis individuals [77]. Moreover, it was reported that rheumatoid arthritis subjects had more missing teeth and twice as likely to have moderate to severe bone loss than non-rheumatoid arthritis subjects [77]. Systemic manifestations of RA are mediated primarily by pro-inflammatory cytokines such as IL-1, IL-6, IL-18, and TNF-α [78], which can induce the production of MMPs leading to further tissue destruction. Study has found that the use of TNF-alpha blockade treatment in RA patients can be beneficial in treating coexisting of periodontitis [79].

### 2.4. Systemic Diseases or Conditions Affecting the Periodontal Supporting Tissues

This particular group of diseases and conditions can affect periodontal tissues causing attachment loss and bone loss independently of plaque-related pathogenesis. These are rare diseases or conditions that primarily include neoplasms (i.e., oral squamous cell carcinoma, odontogenic tumors) or idiopathic diseases (i.e., Langerhans cell histiocytosis, giant cell granulomas, etc.) associated with attachment and tooth loss. Biopsy is usually recommended for the disease diagnosis in order to address the primary cause of periodontal destruction. Since this group of diseases and conditions is independent of plaque accumulation, dental plaque reduction does not result in disease control [23,69].

#### Oral Squamous Cell Carcinoma (OSCC)

Oral squamous cell carcinoma (OSCC) is amongst the most preventable cancers and yet is the sixth most common malignancy globally. Risk factors for OSCC, apart from alcohol and tobacco use, include poor oral hygiene, chronic irritation caused by ill-fitting prosthesis or rough teeth surfaces, poor nutrition, and some chronic sepsis caused by fungi, bacteria, or viruses in the oral cavity [80]. 

Early detection of oral cancer is the key and has a significant impact on patient prognosis. For the early detection of oral cancer, salivary biomarkers and chemokines are the most promising tools. Patients with periodontal disease may be considered to have an increased risk for oral cancer [80]. Education and training of dentists can reduce the consequences of both periodontitis and oral cancer.

## 3. Conclusions

The key components of the periodontal pathophysiology include periodontal ligament (PDL) destruction, bone destruction, gingival inflammation, and bacterial colonization and invasion. Periodontitis is a multifactorial infection whose pathogenesis depends on the complicated interactions between the host immune response and periodontal pathogens. While it is agreed that the primary etiology for periodontal disease is “pathogenic bacteria and its byproduct in a susceptible host”, the understanding of the etiology, disease progression, and disease models of periodontal disease have evolved over the years. Factors such as genetics and the role of the immune system contribute to the individual’s resistance and susceptibility to periodontal disease.

Newman wrote in the 1996 Journal of Dental Research (JDR) article about the connection between periodontal disease and systemic disease “We certainly need to realize that there are links between oral and systemic health and oral and systemic disease. For some the evidence is strong, for others tenuous, and for many indirect but intriguing. Only our research, in collaboration with other medical colleagues in their specialties, will enlighten us” [1]. His words continue to be relevant in the debate on this association in 2020. With the current evidence, the understanding of the pathogenesis of periodontal disease has moved from the consideration of solely a bacterial origin to considering a multifactorial etiology. As our understanding of periodontal disease evolves, our ability to deliver a more “personalized periodontal treatment” improves. Treating periodontal disease, while addressing modifiable risk factors such as smoking, diabetes control, and diet will enhance the quality of patient care. This can possibly lead to more successful outcomes in our treatment of patients with periodontal disease.

## Figures and Tables

**Table 1:** Oral manifestations of Diabetes Mellitus [50].

Periodontal diseases including, gingivitis, varying severity of periodontitis, periodontal abscesses; Salivary and taste dysfunction; Oral infections, fungal and bacterial; Poor wound healing; Non-candida oral soft tissue lesions, including fissured tongue, irritation fibroma and traumatic ulcer; Oral mucosal disease including lichen planus and recurrent aphthous stomatitis; Neuro-sensory oral disorders including oral dysesthesia or burning mouth syndrome; Dental caries and tooth loss.

**Table 2:** Recommended treatment approach for diabetic patients according to the consensus report of the joint European Federation of Periodontology and the American Academy of Periodontology [51].

Inform diabetic patients that periodontal disease and diabetes have a bi-directional relationship; Inform these patients that they should receive a comprehensive oral exam that includes a complete periodontal exam; Provide a presentation of oral health education to the patients; Children and adolescents with diabetes should have an annual oral screening; Diabetic patients are at an increased risk for oral fungal infections and have impaired wound healing.
